# Association with subjective measured physical activity (GPAQ) and quality of life (WHOQoL-BREF) of ageing adults in Hungary, a cross-sectional study

**DOI:** 10.1186/s12889-020-08833-z

**Published:** 2020-08-17

**Authors:** Erzsébet Rétsági, Viktória Prémusz, Alexandra Makai, Csaba Melczer, József Betlehem, Kinga Lampek, Pongrác Ács, Márta Hock

**Affiliations:** 1 Faculty of Health Sciences, Institute of Physiotherapy and Sport Sciences, University of Pécs, 4 Rét str., Pécs, H-7623 Hungary; 2 Faculty of Health Sciences, Doctoral School of Health Sciences, University of Pécs, 4 Vörösmarty str., Pécs, H-7621 Hungary; 3 Faculty of Health Sciences, Institute of Emergency Care and Health Pedagogy, University of Pécs Pécs, 4 Vörösmarty str., Pécs, H-7621 Hungary; 4 Department of Public Health and Health Promotion, Faculty of Health Sciences, Institute of Health Insurance, University of Pécs, 5-7 Mária str., Pécs, H-7621 Hungary

**Keywords:** Physical activity, Aging, Elderly, QoL, GPAQ, WHOQoL-BREF

## Abstract

**Background:**

It is well known that physical activity (PA) has health benefits. This study aimed to examine physical activity carried out by the senior (over 50) participants and its relation to their quality of life (QoL).

**Methods:**

Surveillance of PA and QoL was measured by using questionnaires (GPAQ, WHOQoL-BREF) in this study. Descriptive data were presented as means and standard deviations (SD) for continuous variables and as percentages for categorical variables. Multivariate linear regression analysis was conducted. The significance level was set at *p* ≤ 0.05.

**Results:**

Overall, 250 participants were recruited, the mean age of the study population (*n* = 243) was 70.2 (SD 7.1) years. The results clearly showed that the Hungarian participants aged over 50 years were more likely to do PA if they had university degree and lower age (*p* ≤ 0.001) and used more active transportation (*p* = 0.035) if they had low education. The results of WHOQoL-BREF showed that the Hungarian individuals have better QoL if they have university degree (*p* ≤ 0.001) and lower age (*p* ≤ 0.001). Using multivariate linear regression analysis to examine the effect of PA patterns on QoL adjusted for demographic variables (age, education, BMI, place of living), the result showed significant correlation between WHOQoL-BREF dimensions and GPAQ (*p* ≤ 0.001).

**Conclusion:**

Higher amount of PA among aging population can result in better QoL in all dimensions.

## Background

Nearly one fifth (19.2%) of the European Union (EU) population was aged 65 or more in 2017. The ratio of people aged 80 years or more will probably double by 2080 and it will reach 13% of the whole population [1]. According to data in Hungary, the proportion of people over the age of 60 in 2011 was 23.5% [2]. In 2015, life expectancy in Hungary has increased by almost 4 years since 2000, to 75.7 years, but still lags almost 5 years below the EU average. The gap in life expectancy by socioeconomic status is even larger: Hungarian men with the lowest level of education live on average about 9 years less than men with the highest level of education. Nearly 40% of the overall disease burden in Hungary, as measured by Disability Adjusted Life Years (DALYs) in 2015, can be attributed to unhealthy lifestyles [3]. This is the fourth highest ratio in the EU. Lack of physical activity (PA) is one of the leading behavioral risk factors contributing to DALYs lost in Hungary [4]. The rate of obese adults has doubled by 2014 compared to the 16% of 1988. The growth of the rate can be in correlation with ageing [5]. The ageing process is not uniform across the population due to differences in genetics, lifestyle, and overall health [6]. The chronological age of 65 years as a definition of ‘elderly’ is accepted in general, but the morbidity and mortality rate can begin to increase from 45 years and onwards [7, 8]. Ageing occurs throughout the life course and although there are commonly used definitions of old age, there is no general agreement on the age at which a person becomes old. The changes that constitute and influence ageing are complex [9]. At a biological level, ageing is associated with the gradual accumulation of a wide variety of molecular and cellular damage [10, 11]. Over time, this damage leads to a gradual decrease in physiological reserves, to an increased risk of many diseases, and to a general decline in the capacity of the individual. These changes may coincide with the retirement period.

Physical inactivity is recognized as a global pandemic [12, 13]. Around 31% of the world’s population is not meeting the minimum recommendations for PA, with the highest risk group for inactivity being ageing adults [14]. PA has well documented health benefits such as reducing the risks of developing many non-communicable diseases and of premature mortality [12, 14]. There is also evidence that regular and structured PA enhances mobility, enabling older people to maximize quality of life and independence for longer [15]. The benefits of regular PA include weight control, strengthening of muscles and bones, increases in balance and general physical functioning, and improvements in mental health and health-related quality of life (HRQoL) [16–19]. PA appears to be one of the primary strategies to prevent physiological and cognitive illnesses. Regular PA seems to be a protective factor against genetic and molecular aging and can be associated with longevity [19].

To promote health and prevent diseases, it is important to start these in the earlier period of the people’s life. This study defines the age groups considered for the study as aged 50 years or older. Promoting PA beneficial for health, particularly among older adults aged 50 years and above, has become a challenge for the public health sector specialists in highly developed societies. Actions taken in this field must be based on a thorough examination of health-oriented behaviors of various social groups, particularly on the role of PA.

Therefore, the aim of our study was to examine physical activity carried out by the ageing residents with particular regard to main socio-demographic parameters, like gender, age and education, and the impact on the different domains of the health-related quality of life.

## Methods

### Sample size

Two hundred fifty participants were recruited (convenience sample) at senior’s clubs and retirements associations in Baranya county (Hungary). The data was obtained during face-to-face interviews. We excluded individuals for whom data were missing (*n* = 7). General information on participants was obtained from a paper and pencil questionnaire, including age, gender, educational level and place of living. Educational level was categorized as follows: low (primary schools or less), middle (high school, specialized school and vocational high school), high education (college, university).

Inclusion criteria were age over 50 years; living at home and regularly do not hampered by any illnesses to move and being regularly able to leave the house without physical assistance from another person. Community-dwelling ageing people were excluded, if they were housebound (couldn’t go outside without physical assistance from another person in the past month) or having a cognitive impairment (a diagnosis of dementia or a memory impairment). People having insufficient Hungarian language skills, progressive neurological diseases or medical conditions precluding exercise were also excluded.

### Outcome measures

#### Anthropometric properties

The body mass was measured in kilograms (kg) and the height in meters (m), using a 150 kg capacity digital platform scale (OMRON B511), with a precision of 0.5 kg, and a rigid tape measure of 2 m and 50 cm, respectively. From body mass and height measurements, the Body Mass Index (BMI - kg/m^2^) was calculated (low BMI, if BMI ≤ 18.5 kg/m^2^; normal, if BMI was between 18.5 and 24.9 kg/m^2^; overweight, if BMI was 25 to 29.9 kg/m^2^; obesity, if BMI was 30 to 34.9 kg/m^2^; and obesity grade II was defined as BMI 35 kg/m^2^) [20].

A flexible and inextensible plastic tape, with a precision of 0.1 cm, was used to measure body circumference in centimeters (cm), according to conventional techniques. Waist circumference (WC) was measured at the midpoint between the lower margin of the last palpable rib and the top of the iliac crest based on the WHO STEP wise approach to surveillance (STEPS) protocol, while the hip circumference (HC) was measured around the widest portion of the buttocks in a standing position wearing light clothing and looking straight ahead with arms at the sides and feet adherent to each other. The WHO STEPS protocol suggests that the waist circumference should be measured at the end of a normal expiration, when the lungs are at their functional residual capacity [21]. The measuring tape was kept horizontal during the measurement of the HC and WC. WC and HC were recorded to the nearest cm and waist-hip ratio (WHR) was defined as a ratio of WC to HC. Abnormal WC and WHR were defined with clinically acceptable standard cut-offs. High WC was defined as cut-offs of 80 cm for women and 94 cm for men; high WHR was defined on the basis of World Health Organization criteria as 0.85 for women and 0.90 for men [22].

#### Global physical activity questionnaire

Surveillance of PA in populations is most often undertaken using questionnaires, as these are relatively inexpensive and easy to administer compared to objective measurement techniques. In this study, PA levels were determined by Global Physical Activity Questionnaire (GPAQ).

GPAQ is a suitable and acceptable instrument for monitoring PA in population health surveillance systems [23–25].

The Global Physical Activity Questionnaire was developed by WHO for PA surveillance in countries. This questionnaire was stated as an adequate measurement tool of PA in the elderly [24, 26, 27]. The validated Hungarian version, GPAQ-H was used in the study [28]. It collects information on PA participation in three settings (or domains) as well as on sedentary behavior, comprising 16 questions (P1-P16). The domains are: Activity at work; travel to and from places and recreational activities. For analysis purposes these domains can be further broken down into six different “sub-domains”. These “sub-domains” are: vigorous work (codes P1-P3); moderate work (codes P4-P6); travel (codes P7-P9); vigorous recreation (codes P10-P12); moderate recreation (codes P13-P15); sitting (code P16).

Following the Global Physical Activity Questionnaire (GPAQ) Analysis Guide Vigorous-intensity activities were described as activities that cause large increases in breathing or heart rate like carrying or lifting heavy loads, digging, construction work, running or football. Moderate activities were considered as any activities that cause a small increase in breathing or heart rate such as brisk walking for at least 10 min continuously, i.e. carrying light loads, cycling, swimming or volleyball [29]. Moderate to Vigorous Physical Activity (MVPA) was calculated as the sum of the respective minutes of moderate and vigorous activities of work and leisure time domains. By active transportation GPAQ only describes the duration of the activity without indicating the intensity, as” the usual way of travel to and from places, for example to work, for shopping, to market, to place of worship”. Therefore, the analysis of the test results was completed with a “total” category, which sums up all the minutes spent with PA.

#### WHO’s HRQoL scale (WHOQoL-BREF)

We used the brief version of the WHO’s Quality of Life (QoL) questionnaire (WHOQoL-BREF) from the WHOQoL-100 in this study. The WHOQoL-BREF questionnaire contains two items on overall QoL and general health, and 24 items on different aspects of quality of life that are divided into four domains: physical health with 7 items, psychological health with 6 items, social relationships with 3 items and environmental with 8 items. It has five points Likert-type of scale and higher scores indicated better QoL and the maximum point is 100. It is a valid and reliable measure of the QoL of elderly people [30–32].

### Ethical considerations

All participants were informed of the study’s goals and details, and written informed consent was obtained from participants. The study was conducted in accordance with the World Medical Association’s Helsinki Declaration for Human Studies [33]. This study was approved by the Institutional Review Board of the Faculty of Medicine, University of Pécs, in 2017 (No. 6955).

### Data analysis

Descriptive data were presented as means and standard deviations (SD) for continuous variables and as percentages for categorical variables. To express differences between the socio-demographic, QoL and PA parameters, the independent Mann-Whitney U and Kruskal-Wallis tests was used for the continuous variables and the chi-square test for the categorical variables.

To examine the effect of PA patterns adjusting for demographic variables (age, education, BMI, place of living) we used multivariate linear regression analysis. The significance level was set at *p* < 0.05. For statistical analysis SPSS 24.0 software were used.

## Results

For the present study, a complete data of 243 participants were used in the analyses. Overall, 250 participants were recruited in the current study. The mean age of the study population was 70.20 (SD 7.10) years. Among these 243 participants 38.68% were man and 61.32% were woman. The half of participants had secondary education (49.79%). The majority of the elderly were obese and had higher BMI (26.95 kg/m^2^). Results of the anthropometric characteristics of the genders showed that men have significantly higher BMI (*p* = 0.001) than women. Value of waist circumference was also high in both genders. Results of WHR was 0.91 (men) versus 0.88 (women), which was higher than normal. There were no statistically significant differences in educational attainment, age, and place of living between the genders. The comparative results of the sociodemographic and the anthropometric profile’s characteristics for the study participants are presented in Table 1.

**Table 1:** Descriptive characteristics of the participants by genders

	Total		Female		Male		
	Mean or Frequency	SD or N	Mean or Frequency	SD or N	Mean or Frequency	SD or N	p
**Gender**							
**Female**	61.32	149					
**Male**	38.68	94					
**Age groups**							
**50–59**	20.16	49	18.79	28	22.34	21	0.615
**60–64**	18.52	45	17.45	26	20.21	19	
**65-**	61.32	149	63.76	95	57.45	54	
**Education**							
**Low**	15.64	38	16.11	24	14.89	14	0.966
**Middle**	49.79	121	49.66	74	50.00	47	
**High**	34.57	84	34.23	51	35.11	33	
**Place of living**							
**Urban**	71.60	174	72.48	108	70.21	66	0.702
**Rural**	28.40	69	27.52	41	29.79	28	
**Anthropometric properties**							
**Height (cm)**	168.17	8.78	164.14	7.31	174.50	7.01	**≤0.001**
**Weight (kg)**	76.16	14.50	70.48	11.45	85.33	14.26	**≤0.001**
**Waist circumference (cm)**	95.93	16.08	92.00	17.29	102.73	11.04	**0.003**
**Hip circumference (cm)**	106.91	23.78	107.32	28.19	106.23	13.87	0.091
**Waist to hip ratio**	0.98	0.11	0.88	0.18	0.91	0.16	**≤0.001**
**BMI**	26.95	4.50	26.26	4.75	28.04	3.85	**≤0.001**
**Health status**							
**Average, good, very good (%)**	80.25	202	79.87	119	80.85	76	0.475
**Sport**							
**Does not do any sports regularly**	70.78	172	67.79	101	75.53	71	0.196
**Does sport regularly**	29.22	71	32.21	48	24.47	23	

*BMI* Body Mass Index; *N* number; *SD* standard deviation

One hundred and seventy-two participants (70.78%) were classified as physically inactive. 80.25% of the examined population self-reported ‘average’, ‘good’ and ‘very good’ health status.

The results clearly showed in general that the Hungarian participants aged over 50 years are more likely to do PA if they have lower age and college or university degree (*p* ≤ 0.001) except active transport which was significantly more preferred by individuals with lower educational level (*p* = 0.035). Daily sitting time was similar, gender differences could not be described (Table 2).

**Table 2:** Results of the GPAQ domains by the different demographic variables in ageing adults in Hungary

	min / week		Work time activities	Active transport	Leisure time activities	Daily sitting time	Moderate activities	Vigorous activities	MVPA	Total PA
**Gender**	**Male**	Mean	346.28	169.87	63.14	348.60	253.99	155.43	409.41	579.29
		Std. Deviation	795.93	353.85	170.82	227.00	477.10	492.07	821.24	913.38
	**Female**	Mean	591.51	156.24	72.05	365.77	587.08	76.48	663.56	819.80
		Std. Deviation	4721.54	307.38	163.59	230.28	4737.83	223.66	4770.45	4772.54
	***p*** **value**		0.876	0.982	0.564	0.456	0.966	0.990	0.817	0.957
**Education**	**Low**	Mean	243.55	309.08	25.26	378.37	217.63	51.18	268.82	577.89
		Std. Deviation	385.55	667.28	96.27	267.81	344.87	168.87	436.38	763.94
	**Middle**	Mean	771.12	150.17	63.97	369.09	711.28	123.80	835.08	985.25
		Std. Deviation	5252.92	210.14	190.71	222.85	5261.70	353.71	5309.04	5307.64
	**High**	Mean	215.77	111.11	94.88	336.07	202.56	108.10	310.65	421.76
		Std. Deviation	569.18	183.93	148.87	218.81	303.36	411.57	576.30	631.25
	**p value**		0.322	**0.035**	**< 0.001**	0.477	0.178	0.294	0.183	**< 0.001**
**Age group**	**50–59**	Mean	1597.35	145.82	161.43	324.04	1488.47	270.31	1758.78	1904.59
		Std. Deviation	8215.57	185.05	199.60	202.04	8237.32	616.02	8286.44	8275.17
	**60–64**	Mean	480.44	111.67	50.33	327.56	386.00	144.78	530.78	642.44
		Std. Deviation	779.39	173.80	133.64	234.85	558.52	364.04	789.13	841.84
	**65-**	Mean	139.56	181.73	43.59	380.20	141.24	41.91	183.15	364.89
		Std. Deviation	337.45	389.74	152.69	233.95	282.63	169.09	363.74	557.34
	***p*** **value**		**< 0.001**	0.363	**< 0.001**	0.131	**< 0.001**	**< 0.001**	**< 0.001**	0.515
**Total**		Mean	496.65	161.51	68.60	359.13	458.23	107.02	565.25	726.76
		Std. Deviation	3727.13	325.49	166.13	228.70	3720.47	353.73	3767.25	3776.79

*min/week* minutes per week; *MVPA* moderate to vigorous physical activity; *PA* physical ac tivity; *Std* standard

The results of WHOQoL-BREF showed that the participants had better QoL if they had higher degree (physical health, *p* ≤ 0.001; psychological, *p* ≤ 0.001; social relationships, *p* ≤ 0.001; environment, *p* ≤ 0.001) and lower age (physical health, *p* ≤ 0.001; psychological, *p* ≤ 0.001; social relationship, *p* = 0.002). (Table 3).

**Table 3:** Results of the WHOQoL-BREF domains by the different demographic variables in ageing adults in Hungary

		Physical health	Psychological domain	Social relationships	Environment domain
**Gender**					
**Male**	Mean	58.28	63.07	61.63	61.10
	SD	13.63	15.40	16.92	14.72
**Female**	Mean	58.54	61.84	63.59	62.55
	SD	14.34	15.79	16.79	15.34
**p value**		0.827	0.516	0.196	0.370
**Education**					
**Low**	Mean	54.42	53.89	56.55	56.18
	SD	12.19	11.73	13.83	14.92
**Middle**	Mean	56.21	59.45	59.77	58.90
	SD	13.94	14.93	16.32	14.47
**High**	Mean	63.45	70.26	70.08	69.06
	SD	13.70	14.81	16.46	13.48
**p value**		**< 0.001**	**< 0.001**	**< 0.001**	**< 0.001**
**Age group**					
**50–59**	Mean	66.24	69.33	69.45	64.24
	SD	11.69	13.99	17.42	15.71
**60–64**	Mean	64.47	65.47	65.96	63.09
	SD	12.25	13.93	13.05	15.84
**65-**	Mean	54.05	59.06	59.71	60.91
	SD	13.52	15.74	16.94	14.64
**p value**		**< 0.001**	**< 0.001**	**0.002**	0.354
**Total**					
**Total**	Mean	58.44	62.32	62.83	61.99
	SD	14.04	15.62	16.83	15.09

*SD* standard deviation

There was no significant correlation between work-related PA or active transport and QoL. Although, positive significant association was found between recreational activity and all domains of QoL (≤0.001). Sedentary behavior showed negative relationship with psychological health (*R* = -0.204, *p* ≤ 0.001). If the results were analyzed regarding the intensity and the total amount of PA, positive association could be described with QoL, except the environment domain, as shown in Table 4.

**Table 4:** Correlation between physical activity (measured with GPAQ) and quality of life (measured with WHOQoL-BREF) domains in ageing adults in Hungary

			Physical health	Psychological domain	Social relationships	Environment domain
**GPAQ**	**Work**	R	0.059	0.049	0.066	0.000
		p	0.363	0.445	0.307	0.999
	**Travel to and from places**	R	0.053	0.080	0.000	0.007
		p	0.415	0.216	0.998	0.916
	**Recreational activities**	R	0.343^**^	0.289^**^	0.308^**^	0.239^**^
		p	≤**0.001**	≤**0.001**	≤**0.001**	≤**0.001**
	**Sedentary behaviour**	R	−0.118	−0.204^**^	− 0.120	−0.061
		p	0.067	≤**0.001**	0.062	0.346
	**Moderate PA**	R	0.173^**^	0.180^**^	0.181^**^	0.136^*^
		p	**0.007**	**0.005**	**0.005**	**0.034**
	**Vigorous PA**	R	0.250^**^	0.204^**^	0.186^**^	0.098
		p	≤**0.001**	≤**0.001**	**0.004**	0.127
	**MVPA**	R	0.205^**^	0.202^**^	0.206^**^	0.149^*^
		p	≤**0.001**	**0.002**	**0.001**	**0.020**
	**Total PA**	R	0.215^**^	0.208^**^	0.138^*^	0.115
		p	**0.001**	**0.001**	**0.032**	0.073

**Correlation is significant at the 0.01 level; *Correlation is significant at the 0.05 level (2-tailed)

*R* correlation coefficient; *p* significance (2-tailed)

*MVPA* moderate to vigorous physical activity; *PA* physical activity

In our study, multivariate linear regression analysis was used to examine the effect of PA on QoL adjusted for demographic and anthropometric variables (age, education, BMI, place of living). WHOQoL-BREF measures QoL in four domains, therefore, the analysis was conducted along these four domains.

Negative significant correlation was found between age, BMI and place of living (rural residence) and QoL domains and positive significant relationship between QoL and education.

Time spent with leisure time activity showed significant positive relationship with physical health, psychological domain and social relationships. Active transport (time spent with walking or cycling) correlated only with psychological domain (Table 5).

**Table 5:** Relationship between the WHOQoL-BREF dimensions and demographic variables

		Physical health		Psychological		Social relationships		Environment	
		R^2^ = 0.223, F = 16.513, *p* ≤ 0.001		R^2^ = 0.265, F = 13.995, *p* ≤ 0.001		R^2^ = 0.207, F = 12.250, *p* ≤ 0.001		R^2^ = 0.133, F = 17.639, *p* ≤ 0.001	
**Constant**		72.997	*p* ≤ 0.001	73.716	*p* ≤ 0.001	76.701	*p* ≤ 0.001	60.579	*p* ≤ 0.001
**Beta (p)**	**65+**	−8.741	*p* ≤ 0.001	−4.620	0.013	−4.391	0.034		
	**Education - high**	5.950	0.001	9.688	*p* ≤ 0.001	8.916	*p* ≤ 0.001	9.503	*p* ≤ 0.001
	**Place of living**			−6.525	0.001	−5.434	0.016	−4.660	0.026
	**BMI**	−0.424	0.024	−0.406	0.046	−0.455	0.045		
	**Leisure time activities**	0.012	0.021	0.012	0.024	0.014	0.019		
	**Active transport**			0.006	0.037				

*BMI* Body Mass Index

Excluded variables: gender, Age group 50–59, Age group 60–64, Education – low, Education – middle, GPAQ – Work, GPAQ – Vigorous, GPAQ – Moderate, GPAQ – MVPA, GPAQ – Total, GPAQ – Daily sitting time

## Discussion

One of the most significant challenges may become the lack of PA among the ever-expanding aging population. The lifestyle, more specifically the PA regimen of an individual, changes throughout the lifespan. A significant change in life experienced by individuals older than 50 years could be their transition from employment to retirement, which may influence the health and PA areas. The transition to retirement might lead to a decreased volume of daily PA and at the same time, particularly as a result of an increase in leisure time, the proportion of PA might increase [34, 35]. The health of retirement-age adults has increasing public health importance in Hungary as well, given the demographic trends. By mitigating individuals’ time constraints, retirement provides new opportunities for adopting more physically active lifestyles and increasing the frequency and duration of leisure-time physical exercise [36]. The results of present research can provide further details to PA recommendations with the analysis of PA patterns of aging people in Hungary.

At present, Hungarians are among the most obese nations in Europe, and are in fourth place on a global scale, which may be one of the explanations for poor morbidity and mortality statistics in Hungary [4]. The central obesity is strongly associated with cardiovascular morbidity and mortality [37]. Cardiovascular disease is responsible for more than half of the deaths for people in Hungary. Age-standardized death rates of cardiovascular diseases were more than the double of the EU average in 2014, mainly due to the greater prevalence of smoking and obesity as well [4]. In spite of that, it is well known that regular PA is the most evidence-based strategy for reducing cardiovascular disease risk with aging in both sexes, only total of 29.22% Hungarian participants meet the PA suggestion of ACSM [38]. In a recent analysis of large longitudinal studies by Myers et al. it was found that people who engaged in 150 min moderate intensity (or equivalent) PA per week, had a 31% reduction in mortality compared to those who were less active [39]. On the other hand, one major determinant for overweight/obesity is physical inactivity. Asp and colleagues found strong independent associations between both PA and obesity, and between physical mobility and obesity in the elderly [40].

As an earlier study showed, the environment might influence the PA of individuals. Smaller residential areas indicate a higher proportion of weekly PA [41]. The inhabitants of smaller towns and villages are more likely to achieve PA recommendations [42]. Seniors living in residential areas with ≤100,000 inhabitants are more likely to achieve the walking recommendation in the context of overall walking as well as active transport [43]. In contrast, our findings suggest, that urban living has a positive impact on quality of life, the urban residents have better QoL, than rural residents. In our present study, the amount of urban (71.6%) individuals were more than rural, but this ratio nearly represents the Hungarian (69.5%) distribution [2]. Earlier, the influences of cities’ environmental and built factors were widely demonstrated in the quality of life of aging population [44, 45].

Essential amount of PA can be reported by the aging population in the context of active transport. Based on previous results, PA performed during leisure time and total PA (transportation, work, housework and leisure) can predict the absence of frailty in the elderly [42]. Promoting active transport offers the potential to increase PA levels especially among older adults, since few participate in sport and exercise [18, 46]. Based on our results, the active transportation proved to be one of the good possibilities in elevating the PA level among low-educated older individuals. This information may be relevant for planning services for older people in Hungary.

Education factor is one of the basic determinants of health conscious behavior [47] and of PA [8]. The impacts of education on health are known to derive from knowledge that allow better educated persons to gain access and information on physical activity-promoting resources [48]. Education has a major impact on the quality of life of individuals as revealed by our current findings. According to an earlier study, the amount of occupational PA increases with lower education and higher education may lead to increased leisure PA [49]. Which are in line with the results of our GPAQ analysis, the Hungarian participants aged 50 years and older are more likely to do leisure time activity if they have university/college degree. This finding is consistent with other studies, in which higher education was positively associated with meeting health recommendations and higher education leads to increased leisure PA as well [36]. In our study, results showed, that low-educated individuals participated in active transportation to a greater extent, they walk and use bicycle more frequently. The one third of the Hungarian population (31.7%) have primary-level education [2]. These findings on active transportation could help in the customization of PA programs for the ageing people.

In the present research, people between 50 and 59 years, did more work-related activities, leisure physical activity, vigorous, moderate and total MVPA as well, than older participants. Data from the Baltimore Longitudinal Study on Aging showed remarkable differences in activity levels between the subgroups under 60 and over 75 years [50]. In another examined sample, PA levels decreased also significantly in 10-year-group categories as the age advanced [51].

More than three quarters of the examined population was satisfied with their own health. A previous research showed similar findings, Irish people aged 50+ also reported high life satisfaction [52]. The patients’ feeling of their own good health is an important aspect of positive ageing. It is also a good predictor of other important ageing outcomes, morbidity and mortality.

The QoL of respondents was measured by WHOQoL-BREF questionnaire. In the present research, participants, who had higher degree of education (all dimension of WHOQoL-BREF) and lower age had better QoL, except the environment domain.

Being overweight interfered with QoL equally in both sexes, people with lower BMI had better QoL than individuals with higher BMI [53]. Higher age may be associated with higher BMI [54]. A negative correlation was confirmed in the adult population between BMI and vigorous PA as well. In persons aged ≥50 years with normal BMI the prevalence of a sedentary lifestyle is 9.5%, while in obese individuals it is higher, 10.7% [54, 55]. In the aging population, individuals with normal BMI achieve significantly more steps/day than individuals with overweight and obesity. Weekend days (especially Sunday) were the days with the lowest number of steps in men and women aged ≥50 years with overweight and obesity [56]. Present results indicated an association between increased BMI and decreased QoL in almost all domains (except environmental) of the WHOQoL-BREF.

Our present results showed strong connection between WHOQoL-BREF domains and different demographic variables. We used multivariate linear regression analysis to examine the effect of PA adjusted for demographic and anthropometric (age, education, BMI, place of living) variables. Our results are in line with former findings, i.e. men and women aged ≥65 years report less overall PA compared with men and women aged 50 to 64 years [55]. Professionals should in particular pay attention to physical inactivity (among others) among older people (70 years or older), because it’s relation with quality of life seems to be the strongest [57].

Quality of life and PA data was analyzed by WHOQoL-BREF and GPAQ. Our study showed that significant correlations can be found between the domains of QoL and the PA level. According to the results of this study, recreational activity, vigorous PA, moderate PA and MVPA also improves the respondents’ QoL of physical health domain. According to Puciato et al., the PA level determined the quality of life in older working age populations. Quality of life in the physical dimension was assessed as the highest in the physically most active participants [58].

The present study, as well as some former research, revealed positive relationships between PA and the psychological dimension of quality of life [59]. Some earlier findings suggest that the psychological quality of life was positively affected by moderate- and high-intensity exercises, which was also verified by our present findings [60]. We revealed, that psychological dimension shows inverse connection with sedentary behavior. Sedentary behavior, or time spent non-exercising, reclining and doing seated activities, is emerging as an important public health issue, particularly amongst older people who are the most sedentary group within the population. Several national guidelines recommend a reduction in sedentary behavior for the older adult population [38], although there is little knowledge about factors, that determine sedentary behavior within this population group and how to develop interventions that lead them into changing their behavior.

The present study also confirmed the positive connection between the social relationships’ dimension of quality of life and physical activity, with particular regard to recreational activity, vigorous PA, moderate PA and MVPA. This is highly essential since the first symptoms of body aging may appear in people over 50 years old. These symptoms can have a negative impact on the individual’s functioning in society and interpersonal relations. Thus, in the social relationships area, PA can play preventive roles. Some authors point out that the levels of PA are higher among individuals with enough leisure time (or with sufficient material resources) [61, 62].

The 4th dimension of the WHOQoL-BREF (environment) showed positive correlation with results of recreational activity and MVPA of GPAQ. Among participants who were more active the environment dimension was significantly better.

### Study limitations and strengths

A number of limitations of the study should be mentioned. First, the present study used a relatively small convenience sample of older adults. Second, this study applied a questionnaire-based assessment of PA, which has some limitations. Third, this study did not include complete information from medical records or health examinations about participants.

Consequently, the findings cannot be generalized to older adults with more serious health conditions. Nevertheless, we believe that these limitations do not prevent drawing conclusions from the study.

The strength of the present study is the comprehensive assessment of quality of life in older people. In addition, a large age range of elderly (from 50 years) was studied. Earlier studies on elder populations in bigger countries of Europe and in Hungary clarified, that the social and economic changes related to the transformation from centrally planned to free market economy and gradual convergence with the most highly developed countries can raise question about changes of practice of PA in old age [63, 64]. The important question is how to motivate people to change habits, who are the least motivated because of social and/or economic reasons.

## Conclusion

Although, the sociodemographic and anthropometric characteristics (age, education, place of living and BMI) have an undeniable effect on the quality of life of elderly, PA and particularly leisure time recreational activity plays an important role as well. Elderly, who do more PA have better quality of life in all dimension of WHOQoL-BREF.

## Data Availability

The datasets used and/or analyzed during the current study are available from the corresponding author on reasonable request.

## Abbreviations

ACSM: American College of Sports Medicine
BMI: Body Mass Index
DALYs: Disability Adjusted Life Years
EU: European Union
GPAQ: Global Physical Activity Questionnaire
HC: Hip circumference
HRQoL: Health-Related Quality of Life
min/week: Minutes per week
MVPA: Moderate to vigorous physical activity
N: Number
PA: Physical activity
Std: Standard
SD: Standard deviation
QoL: Quality of Life
WC: Waist circumference
WHR: Waist–hip ratio
WHO: World Health Organization
